# NCX-4040, a nitric oxide-releasing aspirin, sensitizes drug-resistant human ovarian xenograft tumors to cisplatin by depletion of cellular thiols

**DOI:** 10.1186/1479-5876-6-9

**Published:** 2008-02-26

**Authors:** Anna Bratasz, Karuppaiyah Selvendiran, Tomasz Wasowicz, Andrey Bobko, Valery V Khramtsov, Louis J Ignarro, Periannan Kuppusamy

**Affiliations:** 1 Center for Biomedical EPR Spectroscopy and Imaging, Davis Heart and Lung Research Institute, Department of Internal Medicine, The Ohio State University, Columbus, OH 43210, USA; 2 Department of Molecular and Medical Pharmacology, Center for the Health Sciences, University of California School of Medicine, Los Angeles, CA 90095, USA; 3 Comprehensive Cancer Center, The Ohio State University, Columbus, OH 43210, USA

## Abstract

**Background:**

Ovarian carcinoma is the leading cause of mortality among gynecological cancers in the world. The high mortality rate is associated with lack of early diagnosis and development of drug resistance. The antitumor efficacy and mechanism of NCX-4040, a nitric oxide-releasing aspirin derivative, against ovarian cancer is studied.

**Methods:**

NCX-4040, alone or in combination with cisplatin (*cis*-diamminedichloroplatinum, cDDP), was studied in cisplatin-sensitive (A2780 WT) and cisplatin-resistant (A2780 cDDP) cell lines as well as xenograft tumors grown in nude mice. Electron paramagnetic resonance (EPR) was used for measurements of nitric oxide and redox state. Immunoblotting analysis of A2780 cDDP tumor xenografts from mice was used for mechanistic studies.

**Results:**

Cells treated with NCX-4040 (25 μM) showed a significant reduction of cell viability (A2780 WT, 34.9 ± 8.7%; A2780 cDDP, 41.7 ± 7.6%; p < 0.05). Further, NCX-4040 significantly enhanced the sensitivity of A2780 cDDP cells (cisplatin alone, 80.6 ± 11.8% *versus *NCX-4040+cisplatin, 26.4 ± 7.6%; p < 0.01) and xenograft tumors (cisplatin alone, 74.0 ± 4.4% *versus *NCX-4040+cisplatin, 56.4 ± 7.8%; p < 0.05), to cisplatin treatment. EPR imaging of tissue redox and thiol measurements showed a 5.5-fold reduction (p < 0.01) of glutathione in NCX-4040-treated A2780 cDDP tumors when compared to untreated controls. Immunoblotting analysis of A2780 cDDP tumor xenografts from mice treated with NCX-4040 and cisplatin revealed significant downregulation of pEGFR (Tyr845 and Tyr992) and pSTAT3 (Tyr705 and Ser727) expression.

**Conclusion:**

The results suggested that NCX-4040 could resensitize drug-resistant ovarian cancer cells to cisplatin possibly by depletion of cellular thiols. Thus NCX-4040 appears to be a potential therapeutic agent for the treatment of human ovarian carcinoma and cisplatin-resistant malignancies.

## Background

Ovarian carcinoma is the leading cause of mortality among gynecological cancers in the world. The high mortality rate is attributed to the lack of early diagnosis of the malignancy and difficulties associated with treatment. Ovarian cancer is treated using cisplatin drugs; however, relapse of the disease involving a substantial population of cisplatin-resistant cells is commonly observed [1]. The development of drug resistance is a major impediment toward successful treatment of the recurrent cancer [2]. Substantially higher doses of cisplatin and paclitaxel are required to treat the recurrent disease. However, at high doses, these drugs have been known to cause undesirable side effects. For example, cisplatin causes significant nephro- [3], neuro- [4], and ototoxicity [5] while paclitaxel is associated with neurotoxicity and neutropenia [6]. Thus, there have been a number of studies aimed at understanding the causes of drug resistance, so as to develop strategies to overcome or avoid this complication [7-9]. To date, several mechanisms of cisplatin resistance in ovarian cancer cells have been proposed, including decreased cellular uptake and increased cellular efflux of cisplatin [8], inactivation of intracellular cisplatin by glutathione [10-12], and increased levels of DNA repair and DNA tolerance [12,13].

In the past, there have been enormous efforts to develop alternative drugs to treat the recurrent disease. Derivatives of cisplatin, such as carboplatin, oxaliplatin, paclitaxel, doxorubicin, and a variety of alkylating agents have been studied as potential agents to eliminate the resistant cells [1,2]. Also, several strategies were explored to resensitize the refractory cells to established modes of treatment without undesirable side effects. Depletion of cellular thiol levels in the drug-resistant cancer cells is one of the more promising strategies that has been shown to be significantly effective in a number of cases [14-17].

**Figure 1:**
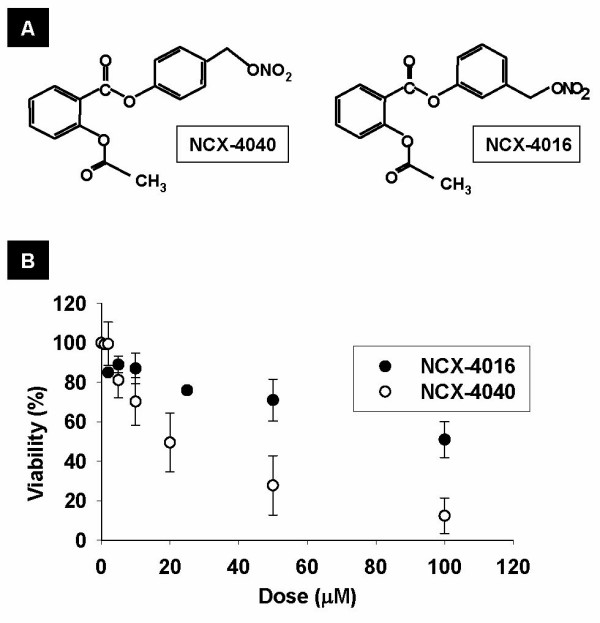
**Comparision of the structure and effect of NCX-4040 and NCX-4016 on cell viability.** (**A**) Molecular structure of NCX-4040 (acetyloxy)benzoic acid 4-(nitrooxymethyl)phenyl ester) and NCX-4016 (2-(acetyloxy)benzoic acid 3-(nitrooxymethyl)phenyl ester). The nitroaspirins consist of acetylsalicylate (aspirin) linked to a spacer moiety by an ester bond. An NO-releasing moiety (-ONO_2_) is attached to the spacer. (**B**) Effect of NCX-4040 and NCX-4016 (25 μM) on the viability of cisplatin-resistant human ovarian cancer cells (A2780 cDDP). The results show a dose-dependent cytotoxic effect, with NCX-4040 demonstrating a substantially higher effect compared to NCX-4016.

Recently, we showed that NCX-4016, a nitro derivative of aspirin, inhibited the proliferation of cisplatin-sensitive as well as cisplatin-resistant human ovarian cancer cell lines *in vitro *[18]. We also showed that NCX-4016, on incubation with the cisplatin-resistant cells, generated sustained levels of nitric oxide (NO) and substantially depleted cellular thiols. In a subsequent report, we further showed that NCX-4016, by itself, was capable of inhibiting the growth of cisplatin-resistant human xenograft tumors in mice [19]. We determined that NCX-4016 induced G_1 _cell-cycle arrest and apoptosis by inhibiting the EGFR/PI3K and STAT3 signaling pathways. Subsequent to the initial reports on NCX-4016, there have been a few reports on NCX-4040 (Figure 1A), a positional isomer of NCX-4016 [20], that demonstrated significant cytotoxic effect on pancreatic [21], bladder [22], and colon cancer [23-25]. It was reported that NCX-4040 was 100 times more potent than NCX-4016 in HT-29 human colon cancer cells [26]. However, its cytotoxic effect on human ovarian cancer has not been investigated. Therefore, the goal of the present work was to study the antitumor efficacy and potential of NCX-4040 to sensitize cisplatin-resistant ovarian cancer cells to cisplatin treatment. The experiments were performed using cisplatin-sensitive (A2780 WT) and cisplatin-resistant (A2780 cDDP) human ovarian cancer cell lines and xenograft tumors. The results showed that administration of NCX-4040 decreased cellular thiol levels, thereby sensitizing the drug-resistant cells to cisplatin. NCX-4040, in combination with cisplatin, inhibited tumor growth by downregulation of EGFR and STAT3 signaling.

## Methods

### Reagents

NCX-4040 (2-(acetyloxy)benzoic acid 4-(nitrooxymethyl) phenyl ester) was obtained from NicOx (Sophia Antipolis, France). GSH (L-glutamyl-L-cysteinylglycine), aspirin (ASA), dimethylsulfoxide (DMSO), buthionine sulfoximine (BSO), S-Nitroso-N-acetylpenicilamine (SNAP), MTT powder (3-(4,5-dimethylthiazol-2-yl)-2,5-diphenyltetrazolium bromide) were obtained from Sigma. 4-amino-5-methylamino-2', 7'-difluorofluorescein diacetate (DAF-FM DA was obtained from Molecular Probes. Cisplatin (*cis*-diamminedichloroplatinum), ammonium iron(II) sulfate hexahydrate, and acetonitrile were purchased from Aldrich. Cell culture medium (RPMI medium 1640), fetal bovine serum (FBS), antibiotics, sodium pyruvate, trypsin, and phosphate-buffered saline (PBS) were purchased from GIBCO/BRL. The imidazoline biradical probes R_1_S-SR_1 _(bis(2,2,5,5-tetramethyl-3-imidazoline-1-oxyl-4-il)disulfide) and R_2_S-SR_2 _(bis((2,2,3,5,5-pentamethyl-1-oxyl-imidazolidinyl-4)-methyl)-disulfide) were synthesized as previously reported [27]. N-methyl-D-glucaminedithiocarbamate (MGD) was synthesized and purified in our laboratory [28]. Polyvinylidene fluoride (PVDF) membrane and molecular weight markers were obtained from Bio-Rad (Hercules, CA). Antibodies for pEGFR (Tyr845, Tyr992), EGFR, pSTAT3 (Tyr705 and Ser727), poly-adenosine diphosphate ribose polymerase (PARP), cleaved caspases-3, Bcl-x_L_, and Bax were purchased from Cell Signaling Technology (Beverly, MA). Antibodies for p53, Survivin, CycD1, and STAT3 were purchased from Santa Cruz Biotechnology (Santa Cruz, CA). Enhanced chemiluminescence (ECL) reagents were obtained from Amersham Pharmacia Biotech (Buckinghamshire, UK).

### Ovarian cancer cell lines

Cisplatin-sensitive (A2780 WT) and cisplatin-resistant (A2780 cDDP) human ovarian cancer cell lines were used. The cisplatin-resistant cell line was originally developed from an *in vivo *tumor model by treating with cisplatin [29]. Cells were grown in RPMI medium 1640 supplemented with 10% FBS, 2% sodium pyruvate, and 1% penicillin/streptomycin. Culture and drug-treatment of cells were carried out at 37°C in an atmosphere of 95% air/5% CO_2_. Cells were routinely trypsinized (0.05% trypsin/EDTA) and counted using an automated counter (NucleoCounter, New Brunswick Scientific Co., Edison, NJ).

### Cell viability assay

Cytotoxicity of the compounds was determined using the conversion of MTT (3-(4,5-dimethylthiazol-2-yl)-2,5-diphenyltetrazolium bromide) to formazan via mitochondrial oxidation. Cells were grown in 75-cm^2 ^flasks to 70% confluence, trypsinized, counted, and plated at a density of 20,000 cells per well in 96 well plates. Cells were incubated overnight and then treated with NCX-4040, ASA and/or cisplatin at variable concentrations for designated lengths of time. All experiments were repeated at least three times. NCX-4040 was dissolved in dimethyl sulfoxide (DMSO) and added into the culture. The final DMSO concentration never exceeded 1%, and this condition was used as the control in each experiment. Cisplatin was dissolved in DMSO (16 mg/ml) and stored at -20°C. The stock solution was diluted to the final concentration prior to each treatment.

The synergism of NCX-4040 and cisplatin was calculated as described [30]. An expected value of cell survival, S_exp_, was defined as the product of the survival observed for NCX-4040 and the survival observed for cisplatin (cDDP): S_exp _= S_NCX-4040_*S_cDDP_. The actual survival observed for the combination treatment was defined as S_obs_. The synergestic ratio, R, was calculated as: R = S_exp_/S_obs_. Synergy was defined for R > 1.

### Measurement of nitric oxide generation using EPR spectroscopy

Generation of NO from NCX-4040 by cells incubated with the compound was measured by EPR spectroscopy using Fe(MGD)_2 _spin trap. The spin trap reacts with NO and forms a stable complex Fe(MGD)_2_-NO which can be detected using EPR. A2780 WT and A2780 cDDP cells were cultured in 75-cm^2 ^flasks. When cells reached 70% confluence, they were trypsinized and resuspended to a final concentration of 20 million cells per milliliter. The cell suspension was added with NCX-4040 (100 μM) and Fe(II)(MGD)_2_, an NO spin-trap prepared in deoxygenated PBS by mixing 1 mM Fe^2+ ^with 5 mM MGD. The NO measurements were performed using a Bruker ER300 X-band EPR spectrometer. The EPR spectra were collected at 20-mW microwave power using the following settings: modulation amplitude, 3 G; modulation frequency, 100 kHz; time constant, 8 ms; scan time, 21 s. SNAP was used as a positive control for NO detection.

### Confocal fluorescence microscopic imaging

Nitric oxide production in A2780 cDDP cells was analyzed using confocal fluorescence microscopic imaging. DAF-FM DA (4-amino-5-methylamino-2',7'-difluorofluorescein diacetate), a green fluorescence probe specific for NO, was added (10 μM) to cells treated with NCX-4040 (100 μM; 0, 1, 2 and 4 h). The probe was diluted in serum and phenol red-free culture medium. Cells were incubated with DAF-FM DA for 30 min at 37°C and then for 15 min without probe to allow complete de-esterification of the intracellular diacetate. Fluorescence measurements were made using a Zeiss LSM 510 multiphoton confocal microscope (excitation, 488 nm; emission, 515 nm). Quantitative analysis of fluorescence intensity was performed using MetaMorph Software.

### Animal housing and preparation

Female 6-week-old BALB/c nude mice were obtained from the National Cancer Institute. The animals were housed five per cage in a climate- and light-controlled room. Food and water were allowed *ad libitum*. All animals were used according to the Public Health Services Policy, the Federal Welfare Act, and ILACUC procedures and guidelines. The mice were anesthetized with ketamine (200 mg/kg b.w.) and xylazine (4 mg/kg b.w.) by intraperitoneal (i.p.) injection. The animals inhaled room room air (21% O_2_) during the *in vivo *spectroscopy, as well as for imaging. The body temperature of the animal was maintained at 37 ± 1°C by an infrared lamp placed just above the animal during the measurements.

### Ovarian cancer tumor xenografts in mice

Cisplatin-sensitive (A2780 WT) and cisplatin-resistant (A2780 cDDP) human ovarian cancer cells (5 × 10^6 ^cells in 60 μl of PBS) were injected subcutaneously (s.c.) into the upper portion of the right hind limb of BALB/c nude mice. Both types of cells grew, *in vivo*, as a solid tumor. The size of the tumor was measured three times per week using a Vernier caliper. The tumor volume was determined from the orthogonal dimensions (d_1_, d_2_, d_3_) using the formula (d_1_*d_2_*d_3_)* π/6.

### In vivo study of NCX-4040 efficiency and redox study

On the 7^th ^day after inoculation, when the tumor size reached approximately 3–5 mm, the mice were subjected to the treatment regime. The mice received i.p. injections of (1) 5 mg/kg b.w. of NCX-4040 alone, daily; (2) 5 mg/kg b.w. of ASA alone, daily; (3) 8 mg/kg b.w. of cisplatin on the 11^th ^day alone; or (4) 5 mg/kg b.w. of NCX-4040 daily, followed by cisplatin treatment on the 11^th ^day (8 mg/kg b.w.). The control group was administered with the vehicle alone (5). At the end of experiment (25^th ^day) the mice were sacrificed. The tumor tissues were then resected and stored in liquid nitrogen up to the time of thiol level determination and immunoblotting analysis.

### Thiol (GSH) assay

Tissue levels of thiols were determined by X-band EPR spectroscopy, using a thiol-specific imidazoline biradical probe [31]. The probe reacts with reduced thiol and shows a characteristic EPR spectrum, which can be quantified by using known amounts of glutathione (GSH). One hundred mg of thawed tissue were homogenized, in 2 ml of PBS, using a tissue homogenizer (PCR Tissue Homogenizing Kit, Fisher Scientific) to obtain a uniform tissue suspension. The tissue homogenate was treated with the thiol-specific label R_1_S-SR_1 _(0.5 mM) at pH 7.0 for 7 min, and the EPR spectra were measured. The concentration of GSH was determined from a standard curve prepared with known concentrations of GSH under similar conditions.

### *In vivo *redox and imaging measurements

Tumor-bearing mice were intratumorally (i.t.) injected with 15 μL of thiol-sensitive probe, R_2_S-SR_2 _[27]. Then EPR measurements were taken immediately and every 1.5 min thereafter for a total of 30 min, during which the signal-decay was observed. The measurements were performed using a home-built L-band (1.2 GHz) EPR spectrometer with a bridged loop-gap resonator [32]. *In vivo *EPR redox images were obtained on the 25^th ^day of tumor growth. Mice were intratumorally injected with 15 μL of R_2_S-SR_2 _[27]. The probe upon reaction with thiols is converted to nitroxide. The total redox information can be obtained from the decay of the nitroxide EPR signal, which depends primary upon reduction factors in the tissue. Mapping of the EPR probe location and its decay was obtained by spatial EPR imaging (EPRI) methods that are well established in our laboratory [32,33]. The EPRI measurements were performed by using a home-built L-band (1.2 GHz) EPR spectrometer with a bridged loop-gap resonator [32].

### Immunoblot analysis

The tumor tissues were homogenized in a lysis buffer containing 10 mM Tris-HCl (pH 7.4), 150 mM NaCl, 1% Triton X-100, 1 mM EDTA, 1 mM EGTA, 0.3 mM phenylmethylsulfonyl fluoride (PMSF), 0.2 mM sodium orthovanadate, 0.5% NP40, 1 μg/ml aprotinin, and 1 μg/ml leupetin. The homogenate was clarified by centrifugation. The protein concentration in the lysates was determined using a Pierce detergent-compatible protein assay kit (Rockford, IL). For immunoblotting, 50 to 100 μg of tissue protein per sample was denatured in 2× SDS-PAGE sample buffer (0.5 mM Tris HCl, 60% glycerol, 10% SDS, and β-mercapto-ethanol, bromophenol blue) and subjected to SDS-PAGE on a 10% or 12% tris-glycine gel. The separated proteins were transferred onto a PVDF membrane. The membrane was blocked with 5% nonfat milk powder (w/v) in Tris-buffered saline Tween-20 (TBST; 10 mM Tris, 100 mM NaCl, 0.1% Tween 20) for 1 h at room temperature or overnight at 4°C. The membranes were incubated overnight with the respective primary antibodies. The bound antibodies were detected with horseradish peroxidase (HRP)-labeled sheep anti-mouse IgG or HRP-labeled donkey anti-rabbit IgG (Amersham Pharmacia Biotech) using an ECL detection system. The ECL image was digitized using a scanner (ScanJet 7400c, Hewlett-Packard, Palo Alto, CA) and quantified using densitometry software (Scion Image v0.4.0.2, Scion, Frederick, MD).

### Statistical analysis

Data were expressed as mean ± SD (*in vitro *study) or mean ± SEM (*in vivo *study). Comparisons among groups were performed by using a Student's *t *test. The significance level was set at p < 0.05.

## Results

### NCX-4040 enhances cisplatin cytotoxicity in ovarian cancer cell lines

**Figure 2:**
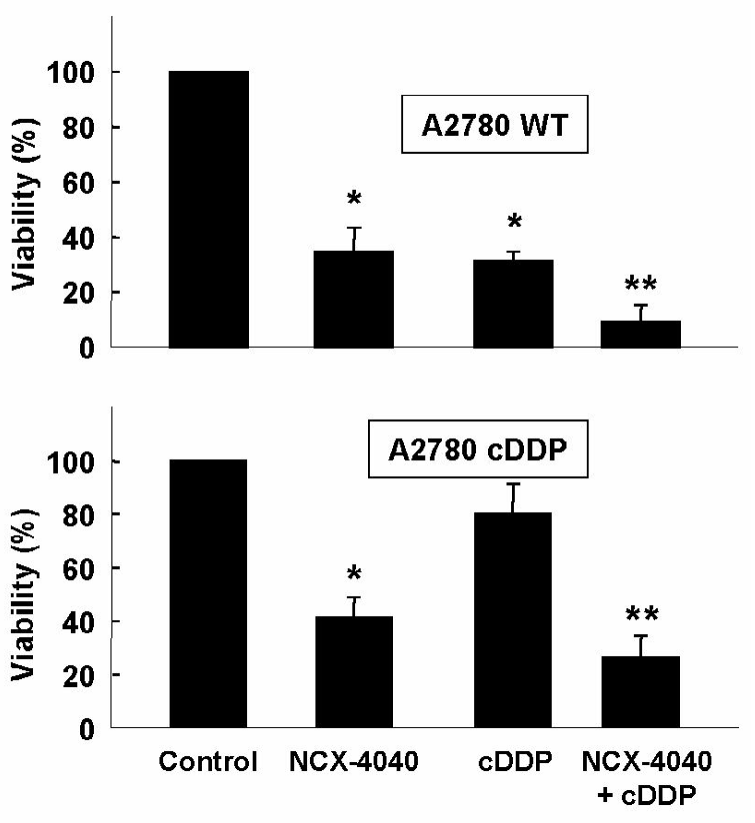
**Effect of NCX-4040 and/or cisplatin on the viability of A2780 WT and A2780 cDDP cell lines.** Cells were incubated with 25 μM of NCX-4040 for 6 h, then with cisplatin for 1 h (IC_67 _dose for A2780 WT) with a 24 h follow up time. Cell viability was evaluated using MTT assay. Data represent mean ± SD obtained from 4 independent experiments and expressed as a percentage of the control cells treated with vehicle (1% DMSO). *p < 0.01 *versus *Control group. **p < 0.05 *versus *cDDP or NCX-4040 group.

NCX-4040 is a positional isomer of NCX-4016 (Figure 1A), which we have previously reported to be cytotoxic to human ovarian cancer cell lines [18]. In order to evaluate the anticancer efficacy of NCX-4040 and to compare with that of NCX-4016, we performed a dose-response study using cisplatin-resistant cell line (A2780 cDDP). As seen in Figure 1B, a dose-dependent decrease of cell viability was observed in both cases. However, NCX-4040 showed a substantial decrease in cell viability when compared to NCX-4016. The cytotoxic effects of NCX-4040, either alone or in combination with cisplatin, on A2780 WT and A2780 cDDP cell lines were determined. Cells were treated with NCX-4040 (25 μM) for 6 h followed by cisplatin (IC_67 _dose for A2780 WT cells) for 1 h with a 24-h follow up time. Cytotoxicity was assessed using an MTT viability assay. The results (Figure 2) showed that NCX-4040 alone significantly decreased the viability of both A2780 WT (34.9 ± 8.7%) and A2780 cDDP (41.7 ± 7.6%) cell lines. Cisplatin (cDDP) alone significantly decreased the viability of A2780 WT (31.5 ± 3.4%), but not A2780 cDDP (80.6 ± 11.8%) cell lines. On the other hand, combination of cisplatin and NCX-4040 was more effective than cisplatin or NCX-4040 alone in decreasing the viability of both A2780 WT (9.4 ± 6.0%) and A2780 cDDP (26.4 ± 7.6%) cell lines. A weak synergism was observed (R = 1.27, see Methods for calculation) between NCX-4040 and cisplatin in the killing of A2780 cDDP cells. The results clearly demonstrated that NCX-4040 was not only cytotoxic, but also capable of sensitizing cisplatin-resistant ovarian cancer cells to cisplatin.

### NCX-4040 generates NO in cells

**Figure 3:**
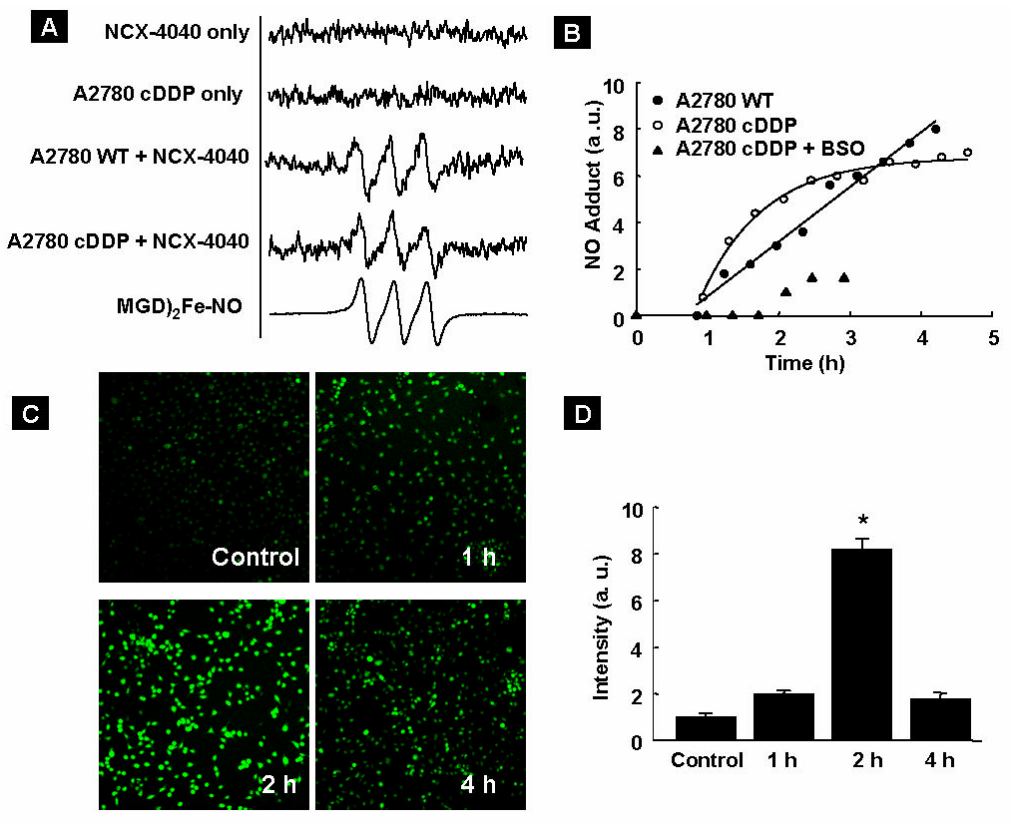
**Generation of nitric oxide (NO) by NCX-4040 in A2780 WT and A2780 cDDP cells.** (**A**) Representative EPR spectra obtained from control (NCX-4040, A2780 cDDP cells) and NCX-4040 treatment (100 μM for both A2780 WT and A2780 cDDP) groups. The cells were incubated with NCX-4040 in the presence of spin trap (MGD)_2_Fe. Also shown is an EPR spectrum of an authentic complex of NO with Fe(MGD)_2 _obtained by adding SNAP (an NO donor) to spin trap. (**B**) Time-course of nitric oxide release by NCX-4040 in A2780 WT, A2780 cDDP, and A2780 cDDP cells treated with 1 mM buthionine sulfoximine (BSO) for 24 h. (**C**) Representative DAF-FM DA fluorescence images of untreated (control) and treated (1, 2 or 4 hours) with NCX-4040 (100 μM). (**D**) Quantitative results on the DAF-FM DA fluorescence data. *p < 0.01 *versus *Control group.

To determine whether NCX-4040 can generate NO in cells, we performed EPR spectroscopic measurements on A2780 WT and A2780 cDDP cells incubated with 100 μM of NCX-4040. Fe(MGD)_2 _was used as a spin-trap for real-time detection of NO. Fe(MGD)_2 _reacts with NO and forms a stable paramagnetic adduct, Fe(MGD)_2_-NO, which can be detected by EPR spectroscopy [34]. NCX-4040 or cells alone in media did not generate any signal (Figure 3A). However, a prominent triplet signal with a hyperfine coupling constant of 12.5 G, characteristic of Fe(MGD)_2_-NO, was observed upon incubation of NCX-4040 with the cells. The authenticity of the spectra was verified using *S*-nitroso-*N*-acetylpenicillamine (SNAP, 100 μM), a known NO-releasing compound. Both cell lines demonstrated a time-dependent increase in the intensity of Fe(MGD)_2_-NO signal (Figure 3B) starting at 40–50 min of co-incubation time. While the rate of NO generation was steady in the case of A2780 WT cells, a rapid increase followed by slow attenuation was observed in the case of A2780 cDDP cells. In order to determine whether or not the increased rate of NO generation in A2780 cDDP cells was due to thiols, we pre-incubated A2780 cDDP cells with 1 mM buthionine sulfoximine (BSO), an inhibitor of glutathione synthesis, for 24 h and then treated with NCX-4040. As seen in Figure 3B, the NO generation was significantly decreased upon BSO treatment, suggesting that the NO generation was mediated by cellular thiols. In contrast, NO generation by SNAP was instant and lasted only for a few minutes (data not shown). We further imaged the intracellular NO generation using DAF-FM DA (Figure 3C). These results shown in Figure 3D clearly demonstrated a sustained generation of NO by NCX-4040 in the cancer cells. Thus, the NO generation by NCX-4040 in cancer cells was low, but persisted for longer periods.

### NCX-4040 inhibits tumor growth in mice

**Figure 4:**
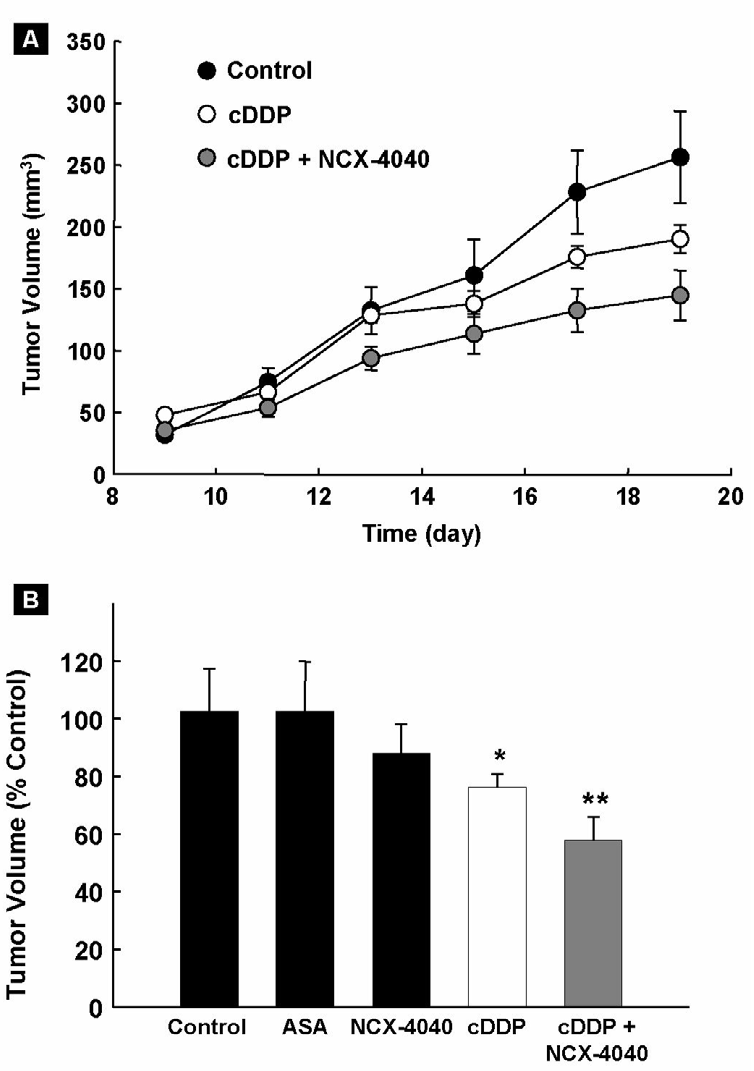
**Effect of NCX-4040 and/or cisplatin (cDDP) on the growth volume of human ovarian cancer xenograft tumor in mice.** Groups of mice (4–6 per group) were inoculated with the A2780 cDDP human ovarian cancer cells in the upper portion of hind leg. Seven days after inoculation, two groups were injected daily (i.p.) with NCX-4040 (5 mg/kg), of which one group received a single i.p. injection of cisplatin on day 11 (8 mg/kg). The third group received single dose of cisplatin on day 11 (8 mg/kg). One group received aspirin (5 mg/kg, daily). Control group received vehicle. (**A**) Tumor growth curve for control, cisplatin, and combination of treatment (NCX-4040 and cisplatin). (**B**) Tumor growth volume data (mean ± SE, expressed as percent of control group) on the 19^th ^day after injection of cancer cells. *p < 0.05 compared to control group. **p < 0.05 versus cDDP group. NCX-4040 and aspirin (ASA), a metabolic product of NCX-4040, showed no significant effect. The results show that pretreatment with NCX-4040 was effective in enhancing the efficacy of cisplatin in inhibiting the growth of cisplatin-resistant ovarian cancer xenografts in mice.

The anti-tumor efficacy of NCX-4040 was studied using A2780 cDDP tumor xenografts in mice. Tumor-bearing animals, on day 7 post-inoculation, were treated with NCX-4040 and/or cisplatin, as described in the Methods section. The tumor volumes were measured every 2 days for 19 days post-inoculation. As shown in Figure 4, NCX-4040 treatment alone did not show any significant reduction in tumor volume (85.8 ± 9.8% *versus *Control on day 19; Figure 4B). Cisplatin (cDDP) treatment alone significantly reduced the tumor growth volume (74.0 ± 4.4% *versus *Control). However, animals treated with a combination of cisplatin and NCX-4040 showed a significant reduction in tumor volume visible from second day after cisplatin treatment and reached 56.4 ± 7.8% *versus *Control on day 19. Aspirin (ASA), a metabolic product of NCX-4040, did not show any significant effect on tumor growth volume (Figure 4B). The results clearly demonstrated that NCX-4040, in combination with cisplatin, was more effective than cisplatin alone in inhibiting the growth of cisplatin-resistant ovarian cancer xenografts in mice.

### NCX-4040 depletes thiol levels in the tumor xenografts

**Figure 5:**
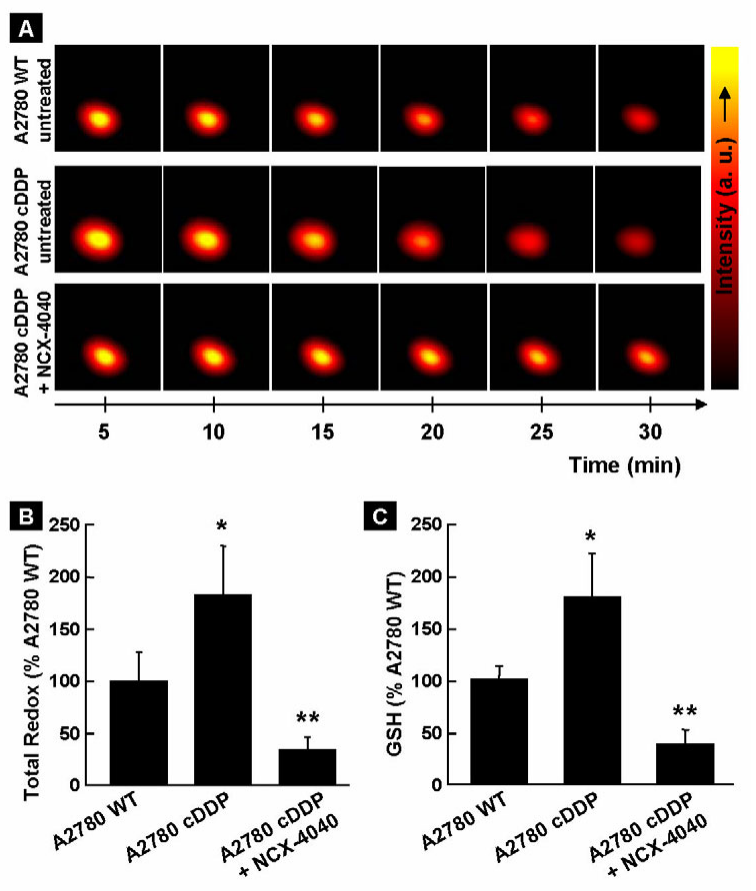
***In vivo*****redox images of ovarian xenograft tumors in mice.** A redox-sensitive probe (R_2_SSR_2_) was injected intratumorally (100 mM; 15 μl), then sequential images were obtained for 30 min. The EPR images show the probe distribution in the tumor, while the tumor itself is not visible. (**A**) Representative images of time-course of redox probe distribution in untreated A2780 WT and A2780 cDDP tumors, as well as in A2780 cDDP tumor treated with NCX-4040 (5 mg/kg, daily). (**B**) Total *in vivo *redox levels expressed as a percent of A2780 WT level. (**C**) Glutathione (GSH) levels in the tumors excised on day 19 post-inoculation. Data represent mean ± SD obtained from 4 mice/group. *p < 0.05 *versus *A2780 WT group. **p < 0.05 *versus *A2780 cDDP group. The results demonstrate that NCX-4040 depleted thiol levels in the cisplatin-resistant tumor.

Since cisplatin-resistance in ovarian cancer is associated with excessive thiol levels, we next wanted to check whether NCX-4040 treatment could modify cellular thiol levels in the solid tumor, *in vivo*. We performed *in vivo *redox imaging of tumor xenografts in mice treated with NCX-4040. As we have reported previously, the *in vivo *redox mapping using EPR imaging enables noninvasive and *real-time *visualization of tumor redox status, which is a measure of total reducing equivalents, including reduced thiols, in the tissue [35]. We used a thiol-specific di-nitroxyl redox probe R_2_S-SR_2_, which on reaction with SH groups breaks down to a mono-nitroxyl with a characteristic triplet EPR signal. The rate of conversion of di- to mono-nitroxyl is a measure of thiol concentration in the tumor. However, in solid tumors, the conversion is very rapid due to excess concentration of thiols compared to the probe. However, the subsequent slower reduction of the EPR-active mono-nitroxyl to EPR-silent hydroxylamine by the cellular reductants including thiols can be monitored and used as a measure of tissue "redox" state. Figure 5A shows typical time-course "redox" images obtained after intra-tumoral injection of R_2_S-SR_2 _(100 mM, 15 μl) in the cisplatin-sensitive (A2780 WT) and cisplatin-resistant (A2780 cDDP) ovarian xenograft tumors treated with NCX-4040. The time-course images showed that the decay of the redox signal was faster in the cisplatin-resistant tumor as compared to that of the cisplatin-sensitive tumor, presumably due to higher cellular reductants in the former cell type. Further, it was observed that NCX-4040 treatment resulted in a substantial slow-down of the redox decay, suggesting that the treatment might have depleted the reductants in the cisplatin-resistant tumor. Quantitative redox data from multiple experiments (Figure 5B) showed that the total redox level was significantly elevated in A2780 cDDP tumor, 182.1 ± 47.4% of A2780 WT tumors' redox. NCX-4040 treatment significantly depressed the total redox level, to 33.3 ± 12.8% (5.5-fold decrease), in A2780 cDDP tumor. We next wanted to measure the modulation of thiol-only levels in the tumors. We performed GSH assay from excised tumor tissues (*in vitro*) using EPR spectroscopy with another thiol-specific probe R_1_SSR_1_, as described in Methods. As seen in Figure 5C, the GSH level was 179.5 ± 42.3% in A2780 cDDP tumor and it was depleted to 38.5 ± 14.1% (a 4.7-fold decrease) after treatment with NCX-4040. The results clearly showed that NCX-4040 depleted thiol level in the cisplatin-resistant tumor.

### NCX-4040 induces apoptosis by downregulating EGFR and STAT3 signaling

**Figure 6:**
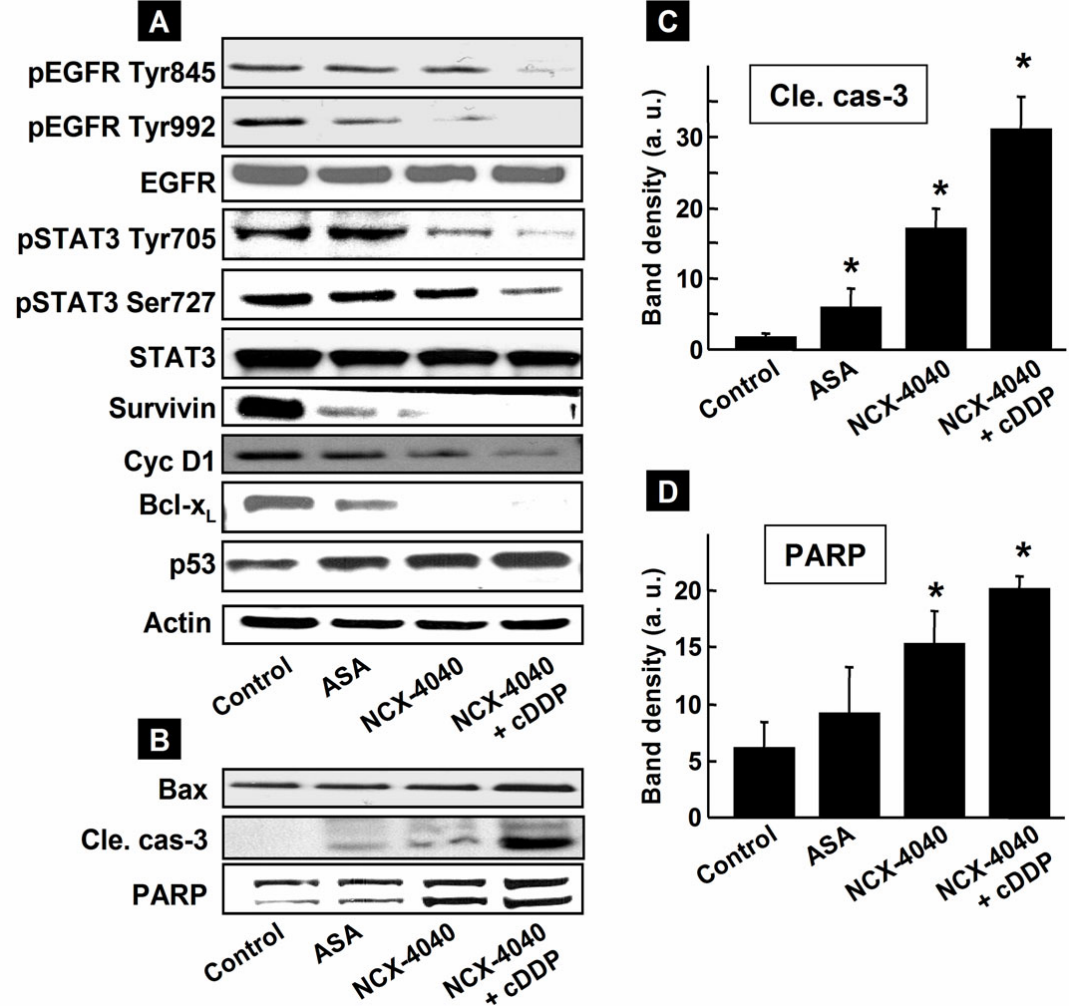
**Immunoblot analysis of tumor tissue.** (**A**) Representative images of EGFR/PI3K and STAT3 signaling pathways. EGFR, pEGFR, pSTAT3, STAT3, cyclin D1, Bcl-x_L_, survivin, p53, actin; (**B**) images for Bax, cleaved caspase-3, and PARP proteins. (**C**) Analysis of cleaved caspase-3 protein band showing an increase of cleaved caspase-3 in cisplatin-resistant tumor treated with aspirin (ASA) or NCX-4040 (5 mg/kg, daily), and NCX-4040 (5 mg/kg, daily) with cisplatin (cDDP, 8 mg/kg, single dose). (**D**) Analysis of PARP protein band showing an increase of PARP in cisplatin-resistant tumor treated with aspirin (ASA), NCX-4040, and NCX-4040 with cisplatin (cDDP). *p < 0.05 *versus *untreated (Control) group.

We recently observed that NCX-4016, an isomeric form of NCX-4040, inhibited tumor growth by modulating EGFR/PI3K and STAT3 signaling pathways [19]. Therefore, we analyzed the EGFR and STAT3 proteins in the A2780 cDDP tumor xenografts from mice treated with NCX-4040 and/or cisplatin. The immunoblots showed that the expressions of pEGFR (Tyr845 and Tyr992) and pSTAT3 (Tyr705, Ser727) were clearly downregulated in the tumors treated with NCX-4040 and cisplatin, when compared to NCX-4040 or aspirin alone groups (Figure 6A). The combination treatment also downregulated the cell-survival proteins including survivin, cyclin D1, Bcl-x_L_, and upregulated the p53 protein. The expression of the proapoptotic protein Bax and apoptotic markers, including cleaved caspase-3 and PARP, were upregulated in the co-treatment group (Figure 6B–D). The results suggested that administration of NCX-4040, in combination with cisplatin, inhibited tumor growth by inducing apoptosis through downregulation of EGFR and STAT3 signaling in the cisplatin-resistant ovarian cancer xenograft in mice.

## Discussion

NCX-4040 belongs to a class of nitric oxide-releasing, non-steroidal anti-inflammatory drugs (NO-NSAID) that were originally developed to overcome the limitation of classical NSAIDs [36]. The NCX-4040 molecule contains an aspirin backbone attached to a -ONO_2 _moiety linked via an aromatic molecular spacer. The -ONO_2 _group, which is responsible for the release of NO, has been shown previously to be required for its anticancer activity [20-22,26]. NCX-4040 is related to NCX-4016 by a positional variation of the -ONO_2 _group; whereas the -ONO_2 _group is at the *para *position in NCX-4040, it is at *meta *position in NCX-4016. The positional isomers, including the *ortho *version (NCX-4060), have been shown to be substantially different in their anticancer efficacies in HT-29 human colon cancer and pancreatic cancer cell lines [20,21,26]. The *ortho*- and *par*a-isomers showed similar IC_50 _values (1–5 μM) for cell-growth inhibition over 72 h, whereas the IC_50 _of the *meta*-isomer was 200 to 500 μM. A structure-activity study of the three isomers also revealed that substituting an aliphatic for the aromatic spacer or removing the -ONO_2 _group profoundly diminished their ability to inhibit cell growth, whereas removal of the acetyl group on the aspirin moiety did not affect cell growth inhibition [20,21,26]. In addition, the three positional isomers exhibited substantial differences in their metabolism and these differences correlated with their differential effects on cancer cell growth, underscoring the importance of positional isomerism in modulating drug effects [20,21,26].

Cisplatin is a widely used chemotherapeutic agent against a number of malignancies, including ovarian cancer [37]. However, development of drug-resistance following prolonged treatment or up on relapse of the disease is an undesirable effect [1]. There is a huge volume of published data that implicates intracellular glutathione as an important factor in cisplatin resistance. Overexpression of glutathione-*S*-transferase enzymes, which catalyze coupling of glutathione to multiple reactive substrates, has also been correlated with low response rate to cisplatin therapy [38-40]. The glutathione-mediated cisplatin resistance has been shown to occur through a number of mechanisms including decreased cellular levels of cisplatin [8], inactivation of cisplatin by formation of glutathione-cisplatin adduct [10-12], increased level of DNA repair and DNA tolerance [12,13], and inhibition of some of the mitochondrial alterations associated with apoptotic commitment [41]. Our results demonstrated a 4.7-fold depression of thiol levels in A2780 cDDP tumor up on treatment with NCX-4040 (Figure 5). This, presumably, was responsible for the resensitization of the tumor to cisplatin, resulting in a significant reduction (56.4%) in tumor volume (Figure 4). The mechanism by which NCX-4040 depletes thiol, however, is not well understood. Gao et al. showed the involvement of GSH and glutathione-S-transferase in the biotransformation of NCX-4040 in the cytosolic fraction of rat liver and colon tissues and in intact HT-29 human colon cancer cells [20]. Other possible mechanisms of thiol depletion might include the thiol-dependent metabolic conversion of the nitrate group (-ONO_2_) to NO in cells and/or formation of nitrosothiols. For example, formation of S-nitrosoglutathione (GSNO) from GSH and NO has been shown to result in a dramatic decrease in GSH levels in activated neutrophils [42]. GSNO itself has been shown to be involved in the modulation of the activity of various enzymes including glutathione-S-transferase [43] and glutathione reductase [44], which is responsible for maintaining high intracellular concentrations of GSH. There is also a recent report which shows that the reaction of other metabolites of nitroaspirin with GSH responsible for thiol depletion [45].

While the cytotoxic effect of NCX-4040 against cisplatin-sensitive (A2780 WT) cells was comparable to that of cisplatin under the conditions used, its effect was significantly higher in the resistant (A2780 cDDP) cells (Figure 2). This indicates that the anticancer potential of NCX-4040 is not inhibited, but in fact potentiated to some extent, by the elevated thiol concentrations in the resistant cells. The substantially higher cytotoxicity induced by the co-administration of NCX-4040 and cisplatin seems to suggest a possible synergistic effect, presumably due to NCX-mediated thiol depletion (Figure 5). Overall, the results demonstrated a dual role for NCX-4040 in the inhibition of drug-resistant ovarian tumor growth by inducing NO-mediated cytotoxicity and resensitizing the refractory cells to cisplatin. Further work is necessary to elucidate their synergistic behavior.

In a previous study, we showed that the cytotoxic effect of NCX-4016 was due to its slow metabolism leading to NO release in cells [18]. We also showed that treatment of cisplatin-resistant ovarian cancer cells with NCX-4016 depleted intracellular thiol levels [18]. In the present work, we have shown thiol content-dependent generation (time-course) NO in cells (Figure 3) and substantial depletion of thiols, as well as total redox, in the tumor tissue (Figure 5). The results clearly demonstrated a cause/effect advantage of thiol utilization/depletion by NCX-4040-mediated NO generation in the cisplatin-resistant ovarian carcinoma. Thus, we have shown that the anti-tumoral and cisplatin-sensitization activities of NCX-4040 were closely linked to its capacity to release NO in the ovarian cancer cells. This is particularly important in the context of a recent report that formation of a quinone methide intermediate from *para*- (NCX-4040) and *ortho*- (NCX-4060) nitroaspirin seems to be responsible for biological activity of these compounds [45-47]. According to this report, the ASA derivative generates the *p*-quinone and *o*-quinone methides, which rapidly react with GSH and activate the antioxidant responsive element (ARE) and NAD(P)H-dependent quinone oxidoreductase (NQO1), probably via reaction with thiol-rich sensor proteins [47]. In general, the metabolism of the meta-form of nitroaspirin (NCX-4016) was slower. NCX-4016 could not form a quinone, but ARE activation and NQO1 induction were observed with slower reaction with thiol-reach proteins [47].

Epidermal growth factor receptor (EGFR) is over-expressed in a wide variety of epithelial malignancies including non-small cell lung, head and neck, colon, and breast cancers [48]. Over-expression and increased activity of EGFR are key characteristics of human tumors and are frequently linked to more aggressive tumor behaviors, including increased proliferation, metastasis, and therapeutic resistance [49]. As such, EGFR is considered to be a key therapeutic target for human cancers [50]. Recent studies have shown that activation of EGFR and STAT3 (signal transducers and activators of transcription 3) confers resistance to chemotherapy-induced apoptosis in epithelial malignancies [49,51]. STAT3 can be activated by various protein tyrosine kinases including Janus tyrosine kinases (JAK) and the proto-oncogene tyrosine kinase (Src), as well as membrane-bound growth factor receptor tyrosine kinases such as EGFR [52]. Many synthetic compounds that inhibit phosphorylated STAT3 and EGFR tyrosine kinase activity have been shown to be effective in preclinical studies and in clinical trials of advance stages of non-small cell lung cancer, breast, and ovarian cancer [53-55]. However, a direct and consistent link between the EGFR and iNOS/NO pathways has not been established. For example, while EGF was shown to induce iNOS expression in normal astrocytes and head and neck squamous cell carcinomas [56], such a regulation was not found in other cell systems. In ovarian carcinoma cells, both exogenously applied NO and endogenously synthesized NO have been shown to inhibit tumor growth [57]. Recently, we have shown that NCX-4016 suppressed the growth of cisplatin-resistant human ovarian xenograft tumor (A2780 cDDP) in mice by inducing G_1 _cell-cycle arrest and apoptosis though inhibition of EGFR/PI3K and STAT3 signaling pathways [19]. In the present study, we also observed that treatment with NCX-4040 led to downregulation of EGFR and STAT3 levels. The blockage of EGFR and STAT3-signaling cascades seemingly increased the levels of p53, Bax and decreased levels of Bcl-x_L_, survivin, and cyclin D1 proteins. This, in turn, inhibited cellular proliferation, and induced apoptosis in cisplatin-resistant tumors. STAT3 is constitutively activated in a variety of tumor cell types including A2780 cDDP ovarian cancer cells [58]. The constitutively active STAT3 has been implicated in the induction of resistance to apoptosis, possibly through the expression of survivin, Bcl-2 and Bcl-x_L _[55]. Bcl-x_L_, which is regulated by both STAT3 Tyr705 and Ser727, is known to be overexpressed in cells resistant to chemotherapy [51,58]. It has been shown that blockage of EGFR and STAT3 cascades resulted in the inhibition of cancer cell proliferation and growth of prostate and liver cancer via cell cycle arrest and apoptosis [50,53].

## Conclusion

The present study demonstrated that NCX-4040 is not only cytotoxic to human ovarian cancer cells, but is also capable of resensitizing drug-resistant, recurrent tumors to cisplatin treatment. NCX-4040 is capable of releasing nitric oxide and depleting thiols in human ovarian cancer cells. NCX-4040 can resensitize and potentiate the anticancer effect of cisplatin through downregulation of EGFR and STAT3 signaling in cisplatin-resistant human ovarian tumor xenografts in mice. Thus, NCX-4040 appears to be a potential therapeutic agent for the treatment of human ovarian carcinoma.

## Abbreviations

cDDP – *cis*-diamminedichloroplatinum; cisplatin; DAF-FM DA – 4-amino-5-methylamino-2', 7'-difluorofluorescein diacetate; EPR – electron paramagnetic resonance; EPRI – electron paramagnetic resonance imaging; GSH – L-glutamyl-L-cysteinylglycine; glutathione; MGD – N-methyl-D-glucamine dithiocarbamate; MTT – 3-(4,5-dimethylthiazol-2-yl)-2,5-diphenyltetrazolium bromide; NCX-4040 – 2-(acetyloxy)benzoic acid 4-(nitrooxymethyl)phenyl ester; nitroaspirin; NCX-4016 – 2-(acetyloxy)benzoic acid 3-(nitrooxymethyl)phenyl ester; nitroaspirin; NSAID – non-steroidal anti-inflammatory drug; SNAP – S-Nitroso-N-acetylpenicillamine; R_1_S-SR_1 _– bis(2,2,5,5-tetramethyl-3-imidazoline-1-oxyl-4-il)disulfide; R_2_S-SR_2 _– bis((2,2,3,5,5-pentamethyl-1-oxyl-imidazolidinyl-4)-methyl)-disulfide.

## Competing interests

The author(s) declare that they have no competing interests.

## Authors' contributions

AB performed the *in vitro and in vivo *experiments, data analysis, and writing of the manuscript. KS was responsible for immunoblotting experiments and data analysis. TW and AB analyzed the redox imaging data. VVK provided the thiol-specific probe. LJI participated in the study design. PK was involved in the study design and write up of the manuscript. All authors read and approved the final manuscript.
